# High expression of angiogenic factor AGGF1 is an independent prognostic factor for hepatocellular carcinoma

**DOI:** 10.18632/oncotarget.22880

**Published:** 2017-12-04

**Authors:** Jianfei Tu, Xihui Ying, Dengke Zhang, Qiaoyou Weng, Weibo Mao, Li Chen, Xulu Wu, Chaoyong Tu, Jiansong Ji, Yuan Huang

**Affiliations:** ^1^ Department of Radiology, Lishui Central Hospital, Lishui Hospital of Zhejiang University, Zhejiang 323000, China; ^2^ Department of Pathology, Lishui Central Hospital, Lishui Hospital of Zhejiang University, Zhejiang 323000, China; ^3^ Department of Hepatobiliary Surgery, Lishui Central Hospital, Lishui Hospital of Zhejiang University, Zhejiang 323000, China

**Keywords:** hepatocellular carcinoma, AGGF1, VEGF, MVD, prognosis

## Abstract

**Background:**

Angiogenesis plays a critical role in tumor growth and metastasis. Angiogenic factor with G patch and FHA domains 1 (AGGF1) has been recently identified as a novel initiator of angiogenesis. However, the function and the prognostic values of AGGF1 in hepatocellular carcinoma remain poorly understood. Our aim is to provide more information to assist design the angiogenesis therapy that targets AGGF1 in HCC.

**Results:**

AGGF1-positive frequency in HCC tissues was significantly higher than in peritumor tissues. The high expression of AGGF1 expression in HCC tissue was well associated with the increased expression of VEGF and the high microvessel density (MVD). AGGF1 expression predicts a poor prognosis and AGGF1 was an independent prognostic factor for DFS.

**Methods:**

The expression levels of AGGF1, vascular endothelial growth factor (VEGF) and microvessel density (MVD) were identified by immunohistochemistry in 79 HCC tumor tissues and 24 corresponding peritumor tissues. The expression level of AGGF1 and MVD were quantified by counting the positively stained endothelial cells in the HCC and the peritumor tissue on the immunohistochemically stained tissue slides. The prognostic value of AGGF1 was evaluated by survival analysis.

**Conclusions:**

Our study shows that AGGF1 is identified as the independent prognostic factor for the disease-free survival (DFS) of patients after the surgical resection. contribute to tumor angiogenesis in HCC, which indicates that AGGF1 may be a new potential therapeutic target for anti-angiogenesis treatment for patients with HCC.

## INTRODUCTION

Hepatocellular carcinoma (HCC) is the most frequent primary liver malignancy, which accounts for the 5th and the 3rd leading cause of death from cancer worldwide in women and men, respectively [1]. The development of cirrhosis is associated with high risk for developing HCC and the etiological factors are various, including hepatitis B virus (HBV), alcohol, other viral hepatitis such as hepatitis C virus (HCV), and nonalcoholic fatty liver disease (NAFLD) [2]. A range of therapies, such as liver transplantation, surgical resection or loco regional therapies including transarterial chemoembolization (TACE), radiofrequency ablation and percutaneous ethanol injection, are used in the management of HCC, however, the prognosis is gloomy with a 5-year survival of 11 % [3–5]. Therefore, identifying novel prognostic and therapeutic biomarkers is very critical for improving the survival of HCC patients.

Angiogenesis is the adequate structure for blood supply, which is playing a critical role in tumor growth and the development of metastasis [6]. Understanding of the basic principles of the biology of angiogenesis is the foundation of developing new prognostic factors and new therapeutic programs.

Angiogenic factor with G patch and FHA domains 1 (AGGF1) was initially identified as a novel angiogenic factor with the ability to the initiation of angiogenesis and maintenance of the vascular network [7] AGGF1 was first characterized as a susceptibility protein in Klippel-Trenaunay syndrome, a congenital vascular disease characterized by malformations of capillary, venous and lymphatic vessels, and bony and soft tissue hypertrophy [8]. In zebrafish embryos, AGGF1 was confirmed involving in the establishment of venous identity [9].

As we known, the process of angiogenesis plays an essential role in tumor growth and metastasis. However, despite the angiogenic activity of AGGF1 in several disease models, its role in the tumor is still limited and controversial. In gastric cancer and HCC patients, expression of AGGF1 was significantly higher than that in adjacent noncancerous samples and increased AGGF1 serves as an unfavorable prognostic factor [10, 11]. In HCC patients, the increased AGGF1 expression is also associated with tumor angiogenesis (microvessel density, MVD). However, the proportion of strong AGGF1 expression was significantly lower in the high-grade urothelial carcinoma group than that in the normal urothelium tissue group [12].

In the present study, the expression of AGGF1, vascular endothelial growth factor (VEGF), and CD34-labeled microvessel density (MVD) in HCC tumor tissues and peritumoral tissues were investigated by immunohistochemistry and evaluated the relationship between the clinical outcome. The distribution of AGGF1 and MVD were visualized by immunofluorescence. Our aim is to provide more information that may be useful for designing more effective angiogenesis therapy that targets AGGF1 in HCC.

## RESULTS

### AGGF1 expression in tumor tissue and the correlation with clinicopathological characteristics

**Figure 1:**
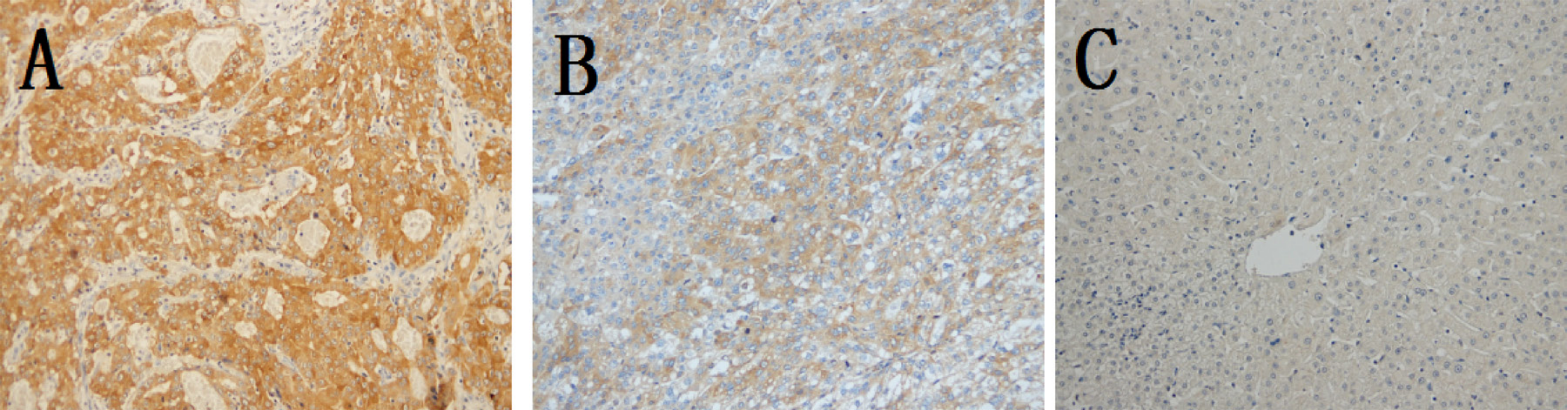
Representative immunohistochemical staining of AGGF1 and higher positive frequency in HCC tissues in HCC tissues Positive staining of AGGF1 was mainly located in the cytoplasm. **(A)**, strongly positive in HCC; **(B)**, positive in HCC; **(C)**, negative in peritumor tissues.

79 HCC tumor tissues and 24 corresponding peritumor tissues, which were randomly selected from the 79 patients as control, were immunohistochemical analyzed to investigate the clinicopathological and prognostic roles of AGGF1 expression. The clinicopathologic characteristics of all patients were summarized in Table 1. As shown in Figure 1, the positive AGGF1 protein staining was mainly in the cytoplasm and could be observed in different tissues with various staining intensities. In tumor tissues, most cases were positive for AGGF1 expression, with a positive rate of 63.3% (50/79), while in peritumor tissues, the positive rate was much lower (37.5%, 9/24). The AGGF1-positive frequency in HCC tissues was significantly higher than in peritumor tissues (P= 0.025, chi-square test).

**Table 1:** Clinicopathologic characteristics of the patients with HCC

Characteristics	Results
Male/female	69/10
Mean age±SD, years	55±12
Median AFP (IQR), ng/ml	94.8, IQR (5.5,408.8)
HBsAg: positive/negative	73/6
ALT: ≤40/>40 U/L	47/32
Cirrhosis: Absent/Present	36/43
Histologic grade: WD/MD or PD	5/74
Tumor size: ≤5/>5 cm	52/27
Tumor number: single/multiple	72/7
BCLC Stage: A/B	41/38

SD: standard deviation; IQR: interquartile range; ALT: alanine aminotransferase; WD: well differentiated; MD: moderately differentiated; PD: poor differentiated; BCLC: Barcelona Clinic Liver Cancer.

The associations of AGGF1 and clinicopathological features were shown in Table 2. Of note, all the patients with multiple tumors belong to positive AGGF1 expression and the positive AGGF1 expression was significantly correlated with tumor number (P=0.035, chi-square test). As for the BCLC stage, positive expression of AGGF1 was significantly correlated with BCLC stage (P=0.020, chi-square test). Interestingly, the other conventional clinicopathological parameters, such as gender (P=0.81), age (P=0.99), AFP (P=0.24), HBsAg (P=0.85), ALT (P=0.72), Cirrhosis (P=0.40), tumor size (P=0.65, all done by chi-square test), none of them was significantly correlated with AGGF1 expression.

**Table 2:** The relationship of AGGF1 expression and clinicopathologic features

Characteristics	AGGF1 expression		P
	-	+-++	
Gender			
Male	25	44	0.81
Female	4	6	
Age			
≤65	22	38	0.99
>65	7	12	
AFP			
≤20	10	24	0.24
>20	19	26	
HBsAg			
Negative	2	4	0.85
Positive	27	46	
ALT			
≤40	18	29	0.72
>40	11	21	
Cirrhosis			
Absent	15	21	0.40
Present	14	29	
Tumor size			
≤5	20	32	0.65
>5	9	18	
Tumor number			
Single	29	43	0.035
Multiple	0	7	
BCLC Stage			
A	20	21	0.020
B	9	29	

### AGGF1 expression correlate with VEGF expression and MVD in HCC tissues

**Figure 2:**
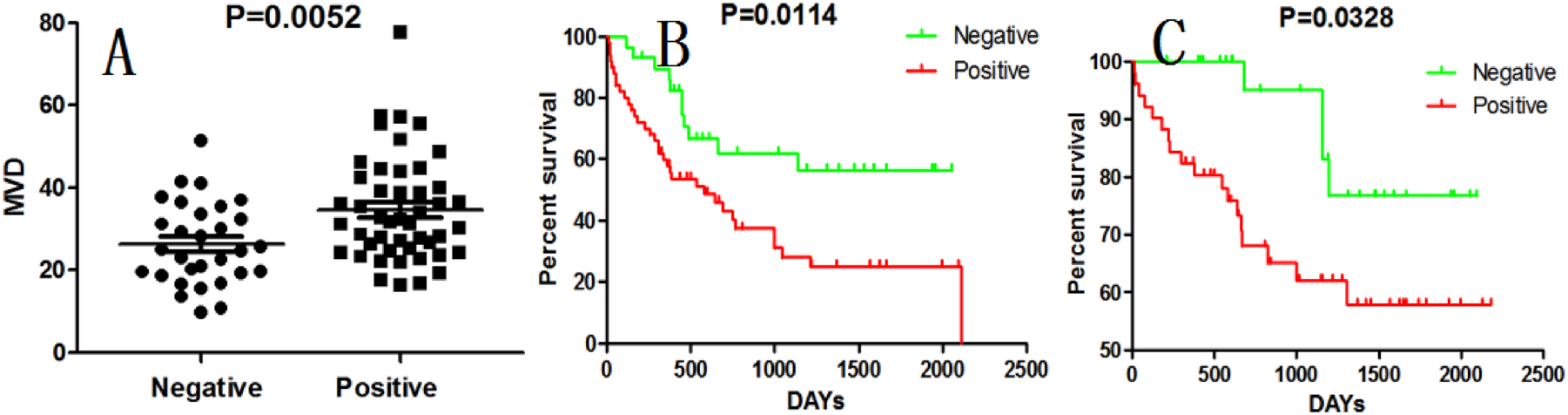
AGGF1 expression in HCC correlates with MVD and prognosis **(A)**, MVD in positive AGGF1 expression patients were significantly higher. **(B)** and **(C)**, patients with AGGF1 expression had a poorer DFS and OS than those with negative ones.

VEGF is a typical angiogenic factor and has a pivotal role in developmental neo-vascularization. To determine the relationship between VEGF and angiogenesis, VEGF and CD34 were detected by Immunohistochemistry. VEGF and MVD were significantly higher in HCC tissue than in peritumor tissue (P=0.045 and P<0.0001, chi-square test). In the total cases, the both AGGF1 and VEGF positive rate was 45.6% (36/79), and the negative rate was 26.6% (15/79). The AGGF1 expression was significantly correlated with VEGF expression (P=0.035, chi-square test). As for MVD, the mean±standard deviation MVD in the AGGF1 positive group was 26±10, that was much higher than in the AGGF1 negative group (35±13, P=0.0052, student’s t-test, Figure 2, A).

### Angiogenesis in HCC tissue may be due to AGGF1 over-expression

**Figure 3:**
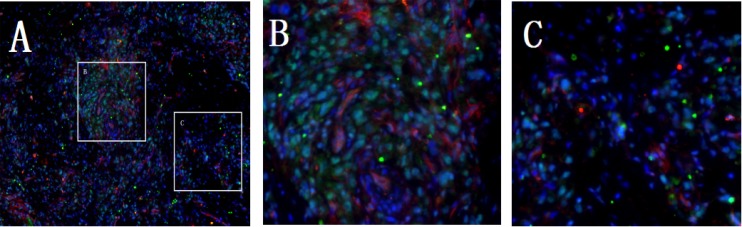
Representative double immunofluorescence in HCC tissue **(A)** the AGGF1 positive staining was in the cytoplasm (green) and CD34^+^ was on the cell surface (red). Sections were counterstained with DAPI (blue). **(B** and **C)** are the local magnify of A.

According to these data above, our evidence indicated that aberrant expression of AGGF1 in HCC tumor tissue was correlated with MVD. Herein, AGGF1 expression and microvessels were visualized in the same field by immunofluorescence. As shown in Figure 3, HCC did overexpress AGGF1, and the microvessels were congested around the over-expressing AGGF1 HCC cells. This phenomenon provided clues that the angiogenesis in HCC tissue was influenced by AGGF1 and it was a local process.

### AGGF1 predicts poor prognosis in HCC patients

Kaplan-Meier method and the log-rank test was used for comparison of outcomes and for MVD, the median value of CD34 positive microvessels was used as a cutoff. Patients with liver cirrhosis or BCLC stage B had a worse prognosis (P = 0.0031 and P=0.0145) and the other conventional clinicopathological features, such as gender, age, AFP levels, HBsAg, ALT, tumor size and tumor number, none of them could predict the DFS (Table 3). Patients with the AGGF1 expression had a poorer DFS and OS than those with negative ones (P = 0.0114 and P=0.0328, Figure 2, B and C). However, VEGF expression and higher MVD had a significantly shorter DFS (P = 0.0175 and P=0.0170), but no association with OS (P = 0.1027 and P=0.1179).

**Table 3:** Univariate and multivariate analysis of factors associated with disease free survival

Variables	Univariate	Multivariate		
	P-value	HR	95% CI	P-value
Male/female	0.7402	-	-	-
Age (years)(≤65 vs.>65)	0.8684	-	-	-
AFP (ng/ml)(≤20 vs.>20)	0.5976	-	-	-
HBsAg	0.7772	-	-	-
ALT(U/L)(≤40 vs. >40)	0.6629	-	-	-
Cirrhosis	0.0031	2.341	1.23-4.46	0.010
Tumor size(cm)(≤5 vs. >5)	0.2691	-	-	-
Tumor number(single vs. multiple)	0.1495	-	-	-
BCLC Stage (A vs. B)	0.0145	2.189	1.19-4.03	0.120
AGGF1	0.0114	2.164	1.09-4.30	0.028
VEGF	0.0175	NA	NA	0.158
MVD	0.0170	NA	NA	0.471

Univariate and multivariate analysis: Cox proportional hazards regression model. HR, Hazard Ratio; NA, not adopted.

In the next step, AGGF1, VEGF and MVD, together with liver cirrhosis and BCLC staging were included in a multivariate Cox proportional hazards analysis and AGGF1 (HR = 2.164; 95% CI: 1.09-4.30; P= 0.028, Table 3), liver cirrhosis and BCLC staging turn out to be independent prognostic factors for DFS. Indicating that patients with AGGF1 expression were nearly 2.164-fold more likely to recurrence than those with AGGF1 negative. As for OS, liver cirrhosis and BCLC staging were the independent prognostic factors (data not shown).

## DISCUSSION

Angiogenesis, which is the adequate structure for blood supply, is the foundation of carcinoma growth and metastasis [6]. HCC is one of the typical hypervascular tumors characterized by neoangiogenesis. A great deal of evidence supports the significance role of angiogenesis in the initiation, development and aggressiveness of HCC [13]. So, understanding the basic principles of the biology of angiogenesis is the key step in in the treatment of HCC.

In this study, we found that AGGF1-positive frequency in HCC tissues was significantly higher than in peritumor tissues. AGGF1 expression was associated with VEGF and MVD and in HCC tissue, the AGGF1 aberrant expressing area was just the MVD high area. Besides, we demonstrated that HCC tumors with AGGF1 expression predicts a poor prognosis and AGGF1 was an independent prognostic factor for DFS.

AGGF1 plays a role in angiogenesis and altered expression of AGGF1 is associated with vascular malformations consistent with Klippel-Trenaunay syndrome (KTS) [8]. Enforced expression of AGGF1 could enhances angiogenesis and improve the blood supply, this may be a treatment or protection for ischemic hindlimbs and myocardial ischemia/reperfusion injury [14, 15]. As for malignancy, the expression of AGGF1 is controversial. In our research, the AGGF1 is up-regulated in HCC tissues than in peritumor tissues. This is consistent with in malignant pleural mesothelioma, gastric cancer and HCC [10, 11, 16]. However, in high-grade urothelial carcinoma, the AGGF1 expression was significantly lower than that in low-grade urothelial carcinoma or in normal control. The probable reason was that hypoxic condition was common in the high-grade urothelial carcinoma and the down-regulating of the AGGF1 protein had an apparent protective role [12]. This inconsistency indicates AGGF1 expression could be altered under different conditions and more studies are still needed.

To determine the role of AGGF1 in angiogenesis, the relationships of AGGF1 expression and VEGF, MVD were explored. As previously reported in gastric cancer and HCC, the positive AGGF1 expression was positively related with aberrant expression of VEGF and higher MVD [10, 11]. β-Catenin has been proved to regulate the transcription of VEGF gene in colon cancer and in human colon cancer cells, by integrative molecular screening, Major et al. found AGGF1 as a nuclear chromatin-associated protein that participates in β-catenin mediated transcription [17]. Actually, deep-sequencing studies have been confirmed that 22% of HCC patients have the mutation of a promoter region of β-Catenin 1 [18]. These findings could, somewhat, explain the relations of AGGF1and VEGF expression. In this study, by immunofluorescence, we firstly visualized AGGF1 expression and microvessels in the same field and found the increased microvessels were congested around the over-expressing AGGF1 HCC cells. We speculate that angiogenesis in HCC tissue is sprouting angiogenesis, a local process but not the influx of circulating endothelial progenitor cells, a systemic vasculogenesis.

The predictive value of AGGF1 in HCC has been reported before [10, 11], its existence in intratumour tissues had a negative impact on the disease-free survival and overall survival of HCC patients and AGGF1 expression was an independent prognostic factor for DFS. However, unlike in the previous studies, our results did not prove AGGF1 expression as independent prognostic factor for OS. Expression of AGGF1 in tumor tissue is relatively different to detect, however, the study of the serum AGGF1 level has not been reported up to now. So the serum AGGF1 level as the prognostic factor should be investigated in the future.

Some limitations should be noted in this paper: 1), this study presents a retrospective study and with relatively small samples; 2), immunohistochemistry was the only method to examine the protein expression levels of AGGF1, VEGF and MVD, lacking gene expression level. And for immunohistochemistry only, there are some factors may influence the staining of intensity and distribution; 3), the exact underlying mechanisms of AGGF1 in angiogenesis was not be clarified.

In conclusion, in this study, we confirmed that AGGF1 was aberrant expressed in HCC tissues and was associated with VEGF and MVD. Positive AGGF1 expression predicts a poor prognosis for DFS and OS and AGGF1 was an independent prognostic factor for DFS. Nowadays, combining antiangiogenic agents, such as sorafenib, represents an effective approach to HCC. Our present results suggest that AGGF1 may contribute to tumor angiogenesis of HCC and could be a new potential therapeutic target for anti-angiogenesis treatment of HCC.

## MATERIALS AND METHODS

### Patients

79 HCC patients, who received curative resection between 2011 and 2015 at the Hepatobiliary Surgery of the Lishui Hospital of Zhejiang University, were obtained written informed consent prior to the research. Tumor tissues were surgically obtained and 24 corresponding peritumor tissues (at least 3cm distant from the tumor site) were randomly selected from the 79 patients as the control. The pathological diagnosis of HCC was confirmed by the standard H.E sections. None of the HCC patients had received any therapies before surgery. Appropriate permission was granted by the ethics committee of the Lishui Central Hospital.

### Immunohistochemistry and immunofluorescence

Immunohistochemistry and immunofluorescence were performed as described in our previous study [19, 20]. Primary antibodies were rabbit anti-AGGF1 (ab203680, 1:200), mouse anti-VEGF (ab1316, 1:150) and mouse anti-CD34 (ab8536, 1:400, all from Abcam, USA). The HRP-conjugated second antibody was from Invitrogen, Carlsbad, CA and the diaminobenzidine was from Beijing Zhongshan Golden Bridge Biotech, China. As for immunofluorescence, the primary antibodies cocktail and the mixture of secondary antibodies were rabbit anti-AGGF1 (1:150), mouse anti-CD34 (1:200) and Alexa Fluor 488-conjugated donkey anti-rabbit, Alexa Fluor 568 donkey anti-goat (all from Invitrogen), respectively.

For the negative control, the primary antibody was carried out with phosphate-buffered saline (PBS).

### Immunohistochemical analysis

The immunohistochemical assessing was performed by two independent, blinded observers (XM Z and WB M). As for AGGF1 and VEGF, the percentage of positive staining cells to the total cells was determined under the 200× high-power magnification. Every sample was randomly selected ten sections for assessing. The expression level of the AGGF1 and VEGF were graded as follows: negative, <10 % of tumor cells with positive staining; positive, ≥10% of tumor cells with positive staining.

MVD was calculated as CD34^+^ vascular endothelial cells. Under 400×high-power magnification, ten areas with the greatest number of distinctly highlighted microvessels were selected for every tissue sample. Vascular endothelial cells or clusters of brown-stained cells were identified as microvessels only if they had clear boundaries with adjacent structures. The average counts of two independent, blinded observers (XM Z and WB M) were used in the following analysis.

### Statistical analysis

Student’s t-tests and Chi-squared test were used to compare AGGF1, VEGF and MVD between tumor tissue and corresponding peritumor tissue. The chi-squared test used to assess the relationship between AGGF1 and the clinicopathological features. The Kaplan-Meier survival analysis and Cox proportional hazards regression model were used for identifying the prognostic factors.

Disease-free survival (DFS) time was defined as the interval between the date of surgery and the date of recurrence. Overall survival (OS) was determined from the date of surgery to the date of death of any cause. It was censored at the time of death or at the last follow-up if the patient remained alive at that time. The Kaplan–Meier model was used for survival analysis. Cox proportional hazards regression model was used for multivariate survival analysis. The analysis was performed using GraphPad Prism 5.01 (GraphPad Software, San Diego, CA, USA) software. P value < 0.05 was taken to indicate statistical significance.

## Abbreviations

AGGF1: Angiogenic factor with G patch and FHA domains 1
HCC: Hepatocellular carcinoma
VEGF: Vascular endothelial growth factor
MVD: Microvessel density
DFS: Disease-free survival
HCV: Hepatitis such as hepatitis C virus
HBV: Hepatitis B virus
NAFLD: Nonalcoholic fatty liver disease
TACE: Transarterial chemoembolization
KTS: Klippel-Trenaunay syndrome
OS: Overall survival
